# Age- and gender-related normal left ventricular deformation assessed by cardiovascular magnetic resonance feature tracking

**DOI:** 10.1186/s12968-015-0123-3

**Published:** 2015-03-10

**Authors:** Florian Andre, Henning Steen, Philipp Matheis, Maria Westkott, Kristin Breuninger, Yannick Sander, Rebekka Kammerer, Christian Galuschky, Evangelos Giannitsis, Grigorios Korosoglou, Hugo A Katus, Sebastian J Buss

**Affiliations:** Department of Cardiology, Angiology and Pneumology, University of Heidelberg, Im Neuenheimer Feld 410, Heidelberg, 69120 Germany; TomTec Imaging Systems, Unterschleißheim, Germany

**Keywords:** Left ventricular function, Feature tracking, Cardiac deformation, Cardiac strain, Strain rates

## Abstract

**Background:**

Assessment of left (LV) ventricular function is one of the most important tasks of cardiovascular magnetic resonance (CMR). Impairment of LV deformation is a strong predictor of cardiovascular outcome in various cardiac diseases like ischemic heart disease or cardiomyopathies. The aim of the study was to provide reference values for myocardial deformation derived from the CMR feature tracking imaging (FTI) algorithm in a reference population of healthy volunteers.

**Methods:**

FTI was applied to standard short axis and 2-, 3- and 4-chamber views of vector-ECG gated CMR cine SSFP sequences of 150 strictly selected healthy volunteers (75 male/female) of three age tertiles (mean age 45.8yrs). Global peak and mean radial, circumferential and longitudinal endo- and myocardial systolic strain values as well as early diastolic strain rates were measured using FTI within a standard protocol on a 1.5T whole body MR scanner.

**Results:**

Global peak systolic values were 36.3 ± 8.7% for radial, −27.2 ± 4.0% for endocardial circumferential, −21.3 ± 3.3% for myocardial circumferential, −23.4 ± 3.4% for endocardial longitudinal and −21.6 ± 3.2% for myocardial longitudinal strain. Global peak values were -2.1 ± 0.5s^−1^ for radial, 2.1 ± 0.6s^−1^ for circumferential endocardial, 1.7 ± 0.5s^−1^ for circumferential myocardial, 1.8 (1.5-2.2)s^−1^ for longitudinal endocardial, 1.6 (1.4-2.0)s^−1^ for longitudinal myocardial early diastolic strain rates. Men showed a higher radial strain than women whereas the circumferential and longitudinal strains were lower resulting in less negative values. Circumferential and longitudinal strain rates were significantly higher in female subjects. Radial strain increased significantly with age whereas the diastolic function measured by the radial, circumferential and longitudinal strain rates showed a decrease.

The coefficients of variation determined in ten further subjects, who underwent two CMR examinations within 12 days, were −4.8% for circumferential and −4.5% for longitudinal endocardial mean strains.

**Conclusions:**

Myocardial deformation analysis using FTI is a novel technique and robust when applied to standard cine CMR images providing the possibility of a reliable, objective quantification of global LV deformation. Since strain values and strain rates differed partly between genders as well as between age groups, the application of specific reference values as provided by this study is recommendable.

## Background

The objective assessment of left ventricular (LV) function is one of the most important tasks of routine cardiovascular magnetic resonance (CMR). Over the years, CMR has emerged as the reference standard for the evaluation of left and right ventricular morphology and function [1]. Especially the early diagnosis of patients with coronary artery disease (CAD) or cardiomyopathies is crucial for the initiation of therapeutic interventions and the reduction of mortality. In recent years, the evaluation of contractile dysfunction with myocardial deformation imaging has been recognized to differentiate various myocardial disorders and has provided important prognostic implications [2-6]. Several advanced diagnostic methods have lately become widely available, thus there is the need for reference values.

Feature tracking imaging (FTI) has recently been introduced for the functional wall motion analysis in CMR cine steady-state free precession (SSFP) images. The feature tracking algorithm is a two-dimensional deformation analysis of the myocardium that was originally designed for post-processing echocardiographic imaging studies, which now has been adapted and applied to standard CMR SSFP images without the need for additional, time-consuming CMR scans or sequences like myocardial tagging, strain-encoding (SENC) or displacement-encoding (DENSE) CMR [7-9]. Furthermore, this novel approach may have potential advantages over existing methods, mainly its broad availability and applicability as well as its vendor independency. Hor and Augustine et al. already showed a good correlation between FTI and MR tagging [10,11]. In addition, the correlation between wall deformation analysis on echocardiography and FTI CMR has shown to be high [12,13]. However, to date, only few data exists on FTI reference values from a large population of precisely characterized healthy volunteers and their relation to age and gender. The aim of this study was to establish reference values of myocardial deformation analysis using FTI, including global peak and mean radial, circumferential and longitudinal systolic strains as well as early diastolic strain rates and to investigate possible age- and gender-related differences.

## Methods

### Study population

One hundred and fifty healthy volunteers (75 women and 75 men, mean age 45.8 + 14.0 years, range 21–71 years) were examined. Volunteers with signs, symptoms or a history of any cardiac disease (including arterial hypertension), cardiovascular, cerebrovascular or relevant noncardiac diseases were excluded. We also excluded all volunteers on regular medication except for contraceptives, chronic thyroid hormone substitution or vitamins. In addition, all volunteers received an oral glucose tolerance test and individuals with impaired glucose tolerance or manifest diabetes mellitus were excluded. An extensive panel of blood tests, including differential blood count, liver enzymes, serum creatinine, thyroid-stimulating hormone, fasting glucose, C-reactive protein, high-sensitivity cardiac Troponin T and N-terminal prohormone brain natriuretic peptide (NT-proBNP), was performed and individuals with abnormal blood test results were excluded. All subjects were screened by clinical history, physical examination, 12-lead electrocardiogram, resting blood pressure measurements and CMR stress tests (adenosine or dobutamine stress). Basic parameters of the study population are provided in Table 1.

For evaluation of age-related characteristics, subjects were classified as “young” (29.3 ± 5.8 years, median 28.5 years, range 21–40 years), as “middle aged” (46.4 ± 3.6 years, median 47.0 years, range 41–54 years) or “old” (61.7 ± 4.2 years, median 60.0 years, range 55–71 years). Each age group consisted of 25 male and 25 female healthy volunteers.

For the assessment of the interstudy reproducibility, ten further subjects (5 male, 5 female) underwent two consecutive CMR examinations within 12 days.

All subjects gave written informed consent. The study was approved by the local institutional ethics committee in accordance with the Declaration of Helsinki. A part of this strictly selected reference population was already included in a prior CMR trial [14].

### Image acquisition

CMR was performed using a clinical 1.5T whole-body MR scanner (Achieva, Philips Medical Systems, Best, The Netherlands) with a dedicated cardiac phased-array receiver coil. All patients were examined in the supine position. For all studies a vector electrocardiogram was used for R-wave triggering. The resting LV function was assessed in cine SSFP images, which were obtained in short axis (SAX) orientation covering the whole LV from base to apex as well as in long axis 2-, 3- and 4-chamber (ch) views. Typical scan parameters were: TE 1.4 ms; TR 2.8 ms; Flip angle 60°; spatial resolution 2.2 mm × 2.2 mm × 8 mm; ≥35 phases per cardiac cycle with a breath-hold time of 7–10 s per image and prospective gating.

### Myocardial deformation imaging

**Figure 1:**
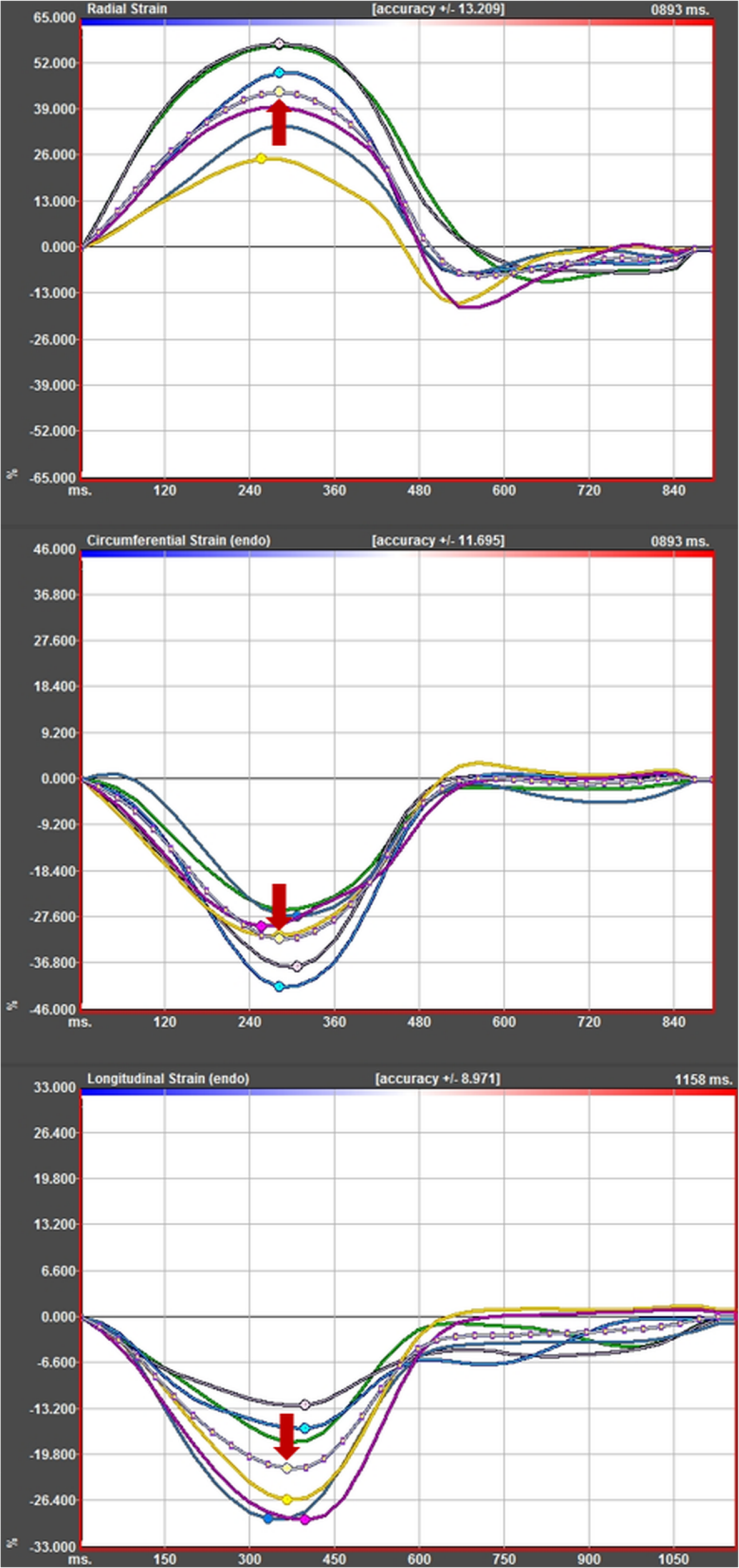
**Representative strain curves.** Exemplary curves showing the radial (top), circumferential (middle) and longitudinal (bottom) strain of the respective cardiac segments. The mean curves are indicated by red arrows.

For strain analysis of the LV short and long axis views, a modified 16-segment LV model according to the standard 17-segment model of the American Heart Association was applied omitting the apical cap. Image analyses were conducted employing the 2D CPA CMR Feature tracking software (TomTec Imaging Systems, Unterschleißheim, Germany). This tool comprises a feature tracking-based analysis software using an algorithm, which has been validated previously in experimental and clinical studies [10,15]. Feature tracking enables the measurement of radial, circumferential and longitudinal strain and strain rates as well as myocardial velocities along user defined endocardial and epicardial borders throughout the cardiac cycle. Endocardial and epicardial borders are initially set in end-diastole of standard cine SSFP short and long axis images. The software algorithm then tracks automatically image features like signal inhomogeneities, tissue patterns of the myocardium or anatomic structures throughout the whole cardiac cycle. The values are derived from the image by comparing the movement of the features in relation to each other along the initially drawn borders. In the case of inadequate tracking after finishing the first measurements, the software allows the editing of the border. The anterior insertion of the right ventricle in short axis views was used to define the segments according to the 16-segment model. The tracking quality was checked using a cine mode, which shows the tracking of the endocardial and epicardial borders throughout the cardiac cycle as well as the resulting strain curves. Segments that did not allow for a reliable tracking were excluded from analysis. In our study, global peak strain values were calculated according to the following approach: on a patient level the peak segmental values of radial, circumferential and longitudinal strains were measured three times and then averaged resulting in global radial, circumferential and longitudinal strain. In order to investigate a practical and fast approach for clinical routine, we additionally calculated the average peak of the mean curve of all segments. This curve represents the average of all segments over the whole cardiac cycle (Figure 1) and provides a mean strain for every image plane, resulting in mean radial, circumferential and longitudinal strain. The early peak diastolic strain rate was obtained from radial, circumferential and longitudinal measurements.

### Reproducibility

Intra- and interobserver variability of the measurements of radial, circumferential and longitudinal endocardial strain were evaluated by two independent blinded observers in 30 randomly selected datasets on a per-segment as well as a per-subject basis. For a detailed analysis of the segmental endocardial strain value reproducibility, the datasets of 30 participants were analyzed without averaging the measurements. The interstudy reproducibility was assessed in the ten subjects who underwent two consecutive CMR examinations within 12 days without averaging the values.

### Statistical analysis

The data are described as mean ± standard deviation or median (interquartile range) as applicable. Continuous variables were compared by Student’s t-test. Otherwise, comparisons between different age-related or gender-related groups were made by Mann–Whitney U-test. Correlation was measured by the Pearson correlation coefficient or the Spearman’s coefficient of rank correlation as appropriate. Intra- and interobserver as well as interstudy variabilities were assessed by coefficients of variation. Age dependency was assessed using a regression analysis if data was distributed normally. Differences were regarded as statistically significant at p < 0.05.

## Results

### Feasibility of radial, circumferential and longitudinal strain measurements

A total of 2400 segments from 150 patients were evaluated for radial, circumferential and longitudinal strain using FTI. At least 96.2% of the segments acquired in short axis views were interpretable. Of the segments obtained in long axis views 95.0% were assessable.

### Strain and strain rate parameters evaluated on a global basis

Global peak systolic values were 36.3 ± 8.7% for radial, −27.2 ± 4.0% for circumferential endocardial, −21.3 ± 3.3% for circumferential myocardial, −23.4 ± 3.4% for longitudinal endocardial and −21.6 ± 3.2% for longitudinal myocardial strain.

Mean values were 34.5 ± 10.2% for radial, −26.5 ± 4.5% for circumferential endocardial, -20.5 ± 3.7% for circumferential myocardial, −21.0 ± 3.9% for longitudinal endocardial and −19.2 ± 3.6% for longitudinal myocardial systolic strain.

The respective segmental and global systolic strain values are given in Tables 2 and 3.

**Table 1:** **Basic parameters of the study population**

	**Mean/Median**	**25% Percentile**	**75% Percentile**	**Range**
**Age (years)**	45.8 ± 14.0	34	58	21-71
**Body weight (kg)**	74.0 ± 12.8	65	82	51-125
**Body height (cm)**	174.0 ± 9.6	167	180	150-198
**Body Mass Index (kg/m** ^**2**^ **)**	24.4 ± 3.1	22.2	26.3	18.9-33.6
**Body Surface Area (m** ^**2**^ **)**	1.9 ± 0.2	1.8	2.0	1.5-2.5
**Systolic blood pressure (mmHg)**	125.5 ± 11.0	117	133	105-160
**Diastolic blood pressure (mmHg)**	75.7 ± 8.6	70	83	55-95
**LDL (mg/dl)**	122.6 ± 31.6	95	143	61-212
**NT-proBNP (pg/ml)**	42.0^†^	27.0	67.5	5-190

Basic parameters of the study population as available.

^†^Median as values were not distributed normally.

**Table 2:** **Reference systolic strain values obtained in short axis views**

**Segment**	**Circumferential endocardial strain**	**Circumferential myocardial strain**	**Radial strain**
**1**	−27.7 ± 8.2	−21.1 ± 6.4	53.2 ± 18.9
**2**	−27.1 ± 7.4	−20.8 ± 6.0	22.1 ± 11.3
**3**	−25.8 ± 7.8	−21.4 ± 6.4	16.3 ± 9.1
**4**	−24.3 ± 8.2	−18.3 ± 6.4	37.1 ± 16.7
**5**	−28.4 ± 8.7	−22.4 ± 7.6	49.4 ± 19.2
**6**	−30.5 ± 9.7	−25.2 ± 8.3	55.3 ± 19.6
**7**	−27.7 ± 7.0	−21.0 ± 6.0	43.4 ± 16.9
**8**	−24.7 ± 6.9	−18.7 ± 5.9	25.2 ± 11.6
**9**	−22.7 ± 5.5	−18.0 ± 4.3	25.2 ± 11.6
**10**	−24.4 ± 6.4	−18.4 ± 5.0	38.6 ± 17.5
**11**	−27.3 ± 7.3	−21.3 ± 6.3	44.1 ± 16.9
**12**	−25.2 ± 7.1	−20.6 ± 6.4	49.1 ± 19.7
**13**	−27.3 ± 7.7	−20.8 ± 6.4	35.2 ± 18.1
**14**	−28.9 ± 8.8	−23.5 ± 7.4	21.6 ± 12.8
**15**	−33.4 ± 9.2	−27.0 ± 8.0	27.0 ± 16.8
**16**	−30.3 ± 8.1	−23.0 ± 6.8	39.1 ± 20.9
**Global peak**	**−27.2 ± 4.0**	**−21.3 ± 3.3**	**36.3 ± 8.7**
**Global mean**	**−26.5 ± 4.5**	**−20.5 ± 3.7**	**34.5 ± 10.2**

All strain values are given in %.

**Table 3:** **Reference systolic strain values obtained in long axis views**

**Segment**	**Longitudinal endocardial strain**	**Longitudinal myocardial strain**
**1**	−23.2 ± 10.1	−21.0 ± 9.6
**2**	−24.9 ± 9.5	−25.1 ± 8.4
**3**	−21.3 ± 8.2	−21.1 ± 7.9
**4**	−24.7 ± 10.8	−24.0 ± 10.0
**5**	−30.9 ± 12.3	−30.8 ± 11.7
**6**	−31.5 ± 10.9	−31.1 ± 10.6
**7**	−28.1 ± 10.3	−25.5 ± 9.4
**8**	−22.2 ± 9.4	−20.7 ± 7.7
**9**	−16.5 ± 8.5	−16.3 ± 8.1
**10**	−17.0 ± 7.8	−15.3 ± 6.9
**11**	−21.1 ± 9.8	−20.7 ± 9.2
**12**	−22.0 ± 11.0	−21.3 ± 10.3
**13**	−27.1 ± 9.1	−22.6 ± 8.0
**14**	−22.6 ± 6.8	−18.4 ± 5.6
**15**	−22.8 ± 9.2	−17.9 ± 7.5
**16**	−18.6 ± 6.8	−14.6 ± 5.5
**Peak 2ch**	−23.8 ± 5.0	−21.1 ± 4.7
**Peak 3ch**	−24.1 ± 5.0	−22.4 ± 4.6
**Peak 4ch**	−20.7 ± 5.1	−19.4 ± 4.9
**Mean 2ch**	−22.0 ± 5.3	−19.4 ± 4.9
**Mean 3ch**	−22.3 ± 5.1	−20.7 ± 4.7
**Mean 4ch**	−18.8 ± 5.4	−17.5 ± 5.0
**Global peak**	**−23.4 ± 3.4**	**−21.6 ± 3.2**
**Global mean**	**−21.0 ± 3.9**	**−19.2 ± 3.6**

All strain values are given in %.

Regarding early diastolic strain rates, the global peak and mean values were −2.1 ± 0.5 s^−1^ and −1.6 (−1.9 to −1.4) s^−1^ for radial, 2.1 ± 0.6 s^−1^ and 1.9 ± 0.6 s^−1^ for circumferential endocardial, 1.7 ± 0.5 s^−1^ and 1.4 ± 0.5 s^−1^ for circumferential myocardial, 1.8 (1.5-2.2) s^−1^ and 1.4 (1.1-1.7) s^−1^ for longitudinal endocardial, 1.6 (1.4-2.0) s^−1^ and 1.3 (1.0-1.6) s^−1^ for longitudinal myocardial early diastolic strain rates.

### Age- and gender-related differences

Global peak and mean systolic strain values as well as early peak diastolic strain rates obtained with FTI were analyzed with regard to age and gender.

The differences between men and women were significant in all assessed strains (p < 0.05) with global peak values of 37.9 ± 8.2% vs. 34.8 ± 8.9% for radial, −26.5 ± 4.2% vs. -27.9 ± 3.7% for circumferential endocardial, −20.4 ± 3.3% vs. -22.2 ± 3.2% for circumferential myocardial, -22.2 ± 3.4% vs. -24.6 ± 2.9% for longitudinal endocardial, −20.4 ± 3.1% vs. -22.9 ± 2.7% for longitudinal myocardial systolic strain. Mean values for men and women were 36.7 ± 10.1% vs. 32.4 ± 9.8% for radial, −25.7 ± 4.8% vs. -27.3 ± 4.0% for circumferential endocardial, -19.6 ± 3.7% vs. -21.4 ± 3.5% for circumferential myocardial, −19.6 ± 3.7% vs. -22.4 ± 3.6% for longitudinal endocardial, -17.7 ± 3.3% vs. -20.7 ± 3.4% for longitudinal myocardial systolic strain. Likewise, the difference between the genders featured significance (all p < 0.05).

Regarding diastolic function, the differences between men and women were significant with global peak values of 2.0 ± 0.6 s^−1^ vs. 2.3 ± 0.6 s^−1^ for circumferential endocardial, 1.5 ± 0.5 s^−1^ vs. 1.8 ± 0.5 s^−1^ for circumferential myocardial, 1.7 (1.4-1.9) s^−1^ vs. 2.0 (1.7-2.3) s^−1^ for longitudinal endocardial, 1.5 (1.3-1.8) s^−1^ vs. 1.8 (1.5-2.2) s^−1^ for longitudinal myocardial early diastolic strain rates (p < 0.05). The global peak radial early diastolic strain rate showed no significant difference between gender groups.

Regarding mean early diastolic strain rates, the values for men and women were 1.7 ± 0.6 s^−1^ vs. 2.0 ± 0.6 s^−1^ for circumferential endocardial, 1.3 ± 0.4 s^−1^ vs. 1.6 ± 0.5 s^−1^ for circumferential myocardial, 1.2 (1.0-1.5) s^−1^ vs 1.5 (1.3-1.9) s^−1^ for longitudinal endocardial and 1.1 (0.9-1.4) s^−1^ vs. 1.4 (1.2-1.8) s^−1^ for longitudinal myocardial early diastolic strain rates. All these gender-related differences were statistically significant (p < 0.05), whereas the mean radial early diastolic strain rate showed no significant differences between gender groups.

**Figure 2:**
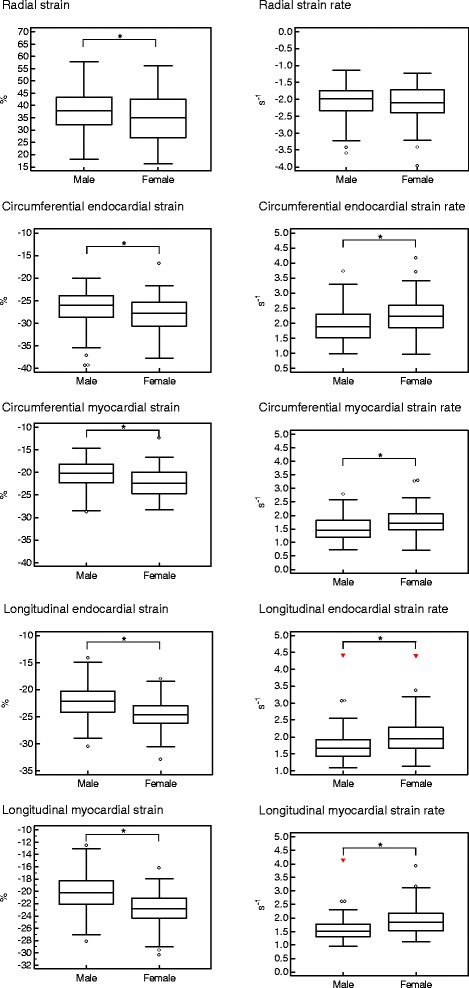
**Gender-related differences in FTI-derived global peak systolic strain values and early diastolic strain rates.** The central box represents the values from the lower to the upper quartile. The middle line shows the median. The whiskers range from the minimum to the maximum value excluding outside values, which are shown as dots, and far out values, which are displayed as triangles. * p < 0.05.

The gender-specific differences are shown in Figure 2.

The global peak and the mean radial systolic strains increased significantly with age (both p < 0.05). Yet, the regression analysis showed no significant age-dependency for the circumferential and longitudinal systolic strains.

The global peak early diastolic radial strain rate showed an age-dependent increase (p < 0.01). Accordingly, the difference between the young and the old group yielded significance for the mean radial early diastolic strain rate (−1.7 s^−1^ vs. -1.6 s^−1^; p < 0.05).

The global peak and mean circumferential endocardial as well as myocardial early diastolic strain rates decreased significantly with age (all p < 0.01).

Regarding the longitudinal early diastolic strain rates, the group of the young subjects had significant higher global peak and mean values of endocardial (1.9 s^−1^ vs. 1.8 s^−1^ vs. 1.7 s^−1^ and 1.6 s^−1^ vs. 1.4 s^−1^ vs. 1.2 s^−1^; both p < 0.05) as well as myocardial (1.8 s^−1^ vs. 1.6 s^−1^ vs. 1.5 s^−1^ and 1.4 s^−1^ vs. 1.2 s^−1^ vs. 1.1 s^−1^, both p < 0.05) strain rates.

**Figure 3:**
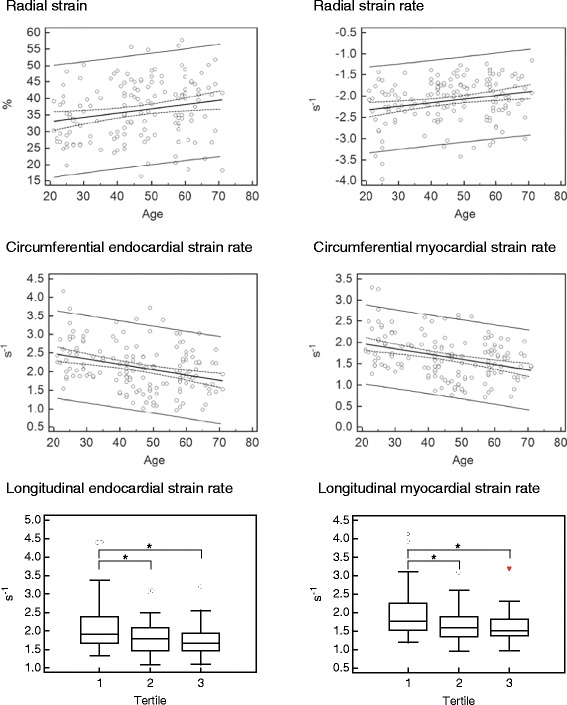
**Age-related differences in FTI-derived global peak systolic strain values and early diastolic strain rates.** Scatter diagrams with regressions lines and box-and-whisker plots as applicable. The scatter diagram includes the regression line with the respective 95% confidence curves as well as the 95% prediction curves. The central box of the box-and-whisker plot represents the values from the lower to the upper quartile. The middle line shows the median. The whiskers range from the minimum to the maximum value excluding outside values, which are shown as dots, and far out values, which are shown as triangles. * p < 0.05.

The age-dependencies of the systolic strain values and early diastolic strain rates are displayed in Figure 3.

Age- and gender-specific reference values for systolic strains are provided in the Tables 4, 5, 6, 7 and 8.

**Table 4:** **Radial systolic strain values**

	**Men**				**Women**			
	**Tertile**				**Tertile**			
**Segment**	**1**	**2**	**3**	**All**	**1**	**2**	**3**	**All**
**1**	52.5 ± 16.1	49.3 ± 16.1	52.9 ± 18.0	51.6 ± 16.6	53.4 ± 20.7	58.0 ± 22.0	53.2 ± 20.9	54.9 ± 21.0
**2**	19.7 ± 7.2	17.8 ± 7.8	20.6 ± 9.7	19.3 ± 8.3	25.7 ± 9.8	24.6 ± 13.3	24.0 ± 16.3	24.8 ± 13.2
**3**	15.5 ± 12.1	20.1 ± 7.9	16.9 ± 10.1	17.5 ± 10.2	15.5 ± 5.9	16.5 ± 8.9	13.2 ± 7.8	15.1 ± 7.6
**4**	34.9 ± 16.6	43.8 ± 13.1	38.9 ± 20.2	39.2 ± 17.0	32.4 ± 16.6	37.3 ± 12.2	35.4 ± 19.4	35.0 ± 16.2
**5**	49.5 ± 17.6	53.8 ± 16.3	51.5 ± 23.0	51.6 ± 19.0	42.6 ± 17.9	53.5 ± 20.6	45.6 ± 18.2	47.2 ± 19.3
**6**	56.2 ± 15.6	53.5 ± 13.3	57.3 ± 21.9	55.7 ± 17.0	50.7 ± 22.7	58.6 ± 22.9	56.0 ± 20.5	55.0 ± 22.0
**7**	42.3 ± 15.6	46.5 ± 17.6	46.3 ± 19.6	45.0 ± 17.6	37.7 ± 13.1	43.7 ± 17.7	43.9 ± 17.0	41.7 ± 16.1
**8**	24.1 ± 11.7	22.7 ± 10.2	23.5 ± 10.2	23.4 ± 10.6	27.0 ± 8.8	26.2 ± 10.6	27.7 ± 16.5	27.0 ± 12.3
**9**	25.6 ± 10.9	29.9 ± 12.7	23.9 ± 11.8	26.5 ± 11.9	20.7 ± 8.2	26.4 ± 12.4	24.9 ± 12.2	24.0 ± 11.2
**10**	35.7 ± 15.5	47.5 ± 14.4	35.6 ± 18.4	39.5 ± 16.9	31.4 ± 11.5	41.5 ± 19.1	40.1 ± 21.3	37.7 ± 18.1
**11**	43.9 ± 16.0	48.4 ± 15.4	47.4 ± 18.6	46.5 ± 16.6	34.8 ± 14.9	43.3 ± 16.8	47.5 ± 17.1	41.8 ± 16.9
**12**	51.4 ± 19.9	57.7 ± 20.2	52.7 ± 21.8	54.0 ± 20.5	33.9 ± 14.3	47.2 ± 17.1	52.6 ± 17.1	44.4 ± 17.8
**13**	30.6 ± 17.6	39.5 ± 22.2	41.0 ± 15.5	37.0 ± 19.0	32.9 ± 19.3	35.4 ± 15.7	32.0 ± 16.7	33.4 ± 17.1
**14**	19.4 ± 9.2	23.0 ± 14.9	23.5 ± 12.6	22.0 ± 12.4	19.6 ± 9.6	21.0 ± 13.1	23.3 ± 16.4	21.3 ± 13.2
**15**	27.0 ± 13.1	35.3 ± 18.8	34.5 ± 17.2	32.3 ± 16.7	16.3 ± 10.6	20.7 ± 13.4	27.9 ± 18.4	21.6 ± 15.1
**16**	40.1 ± 19.8	49.3 ± 25.8	45.0 ± 20.2	44.8 ± 22.1	27.1 ± 16.4	33.5 ± 19.9	39.7 ± 15.8	33.4 ± 18.0
**Global peak**	**35.5 ± 7.3**	**39.9 ± 7.8**	**38.2 ± 9.1**	**37.9 ± 8.2**	**31.4 ± 7.6**	**36.6 ± 8.6**	**36.5 ± 9.8**	**34.8 ± 8.9**
**Global mean**	**33.1 ± 7.9**	**39.0 ± 9.7**	**37.9 ± 11.7**	**36.7 ± 10.1**	**28.4 ± 8.1**	**34.4 ± 9.7**	**34.5 ± 10.6**	**32.4 ± 9.8**

All strain values are given in %.

**Table 5:** **Circumferential endocardial systolic strain values**

	**Men**				**Women**			
	**Tertile**				**Tertile**			
**Segment**	**1**	**2**	**3**	**All**	**1**	**2**	**3**	**All**
**1**	−28.2 ± 7.6	−25.5 ± 7.7	−27.6 ± 8.5	−27.1 ± 7.9	−26.2 ± 8.3	−29.0 ± 5.9	−29.8 ± 10.3	−28.3 ± 8.4
**2**	−25.8 ± 7.1	−27.4 ± 6.9	−24.8 ± 5.8	−26.0 ± 6.6	−28.1 ± 7.0	−26.3 ± 8.7	−30.3 ± 8.0	−28.2 ± 7.9
**3**	−25.7 ± 6.5	−23.3 ± 6.9	−25.5 ± 8.9	−24.8 ± 7.4	−26.0 ± 6.9	−26.5 ± 9.8	−27.5 ± 7.7	−26.7 ± 8.1
**4**	−23.5 ± 8.1	−21.0 ± 6.1	−28.1 ± 9.9	−24.2 ± 8.6	−22.8 ± 7.2	−25.6 ± 7.9	−24.9 ± 8.2	−24.4 ± 7.8
**5**	−27.3 ± 9.1	−24.8 ± 8.9	−31.3 ± 7.7	−27.8 ± 8.9	−26.5 ± 6.8	−28.3 ± 7.9	−32.6 ± 10.0	−29.0 ± 8.5
**6**	−28.7 ± 7.6	−24.9 ± 8.5	−34.2 ± 8.8	−29.2 ± 9.0	−27.9 ± 8.9	−33.7 ± 9.2	−34.1 ± 11.4	−31.8 ± 10.2
**7**	−26.9 ± 5.5	−24.3 ± 6.2	−28.7 ± 7.2	−26.7 ± 6.5	−27.1 ± 8.1	−29.6 ± 6.6	−29.4 ± 7.5	−28.7 ± 7.4
**8**	−24.0 ± 6.3	−24.8 ± 7.4	−24.0 ± 8.3	−24.2 ± 7.2	−23.7 ± 4.6	−25.0 ± 7.1	−26.5 ± 7.6	−25.1 ± 6.6
**9**	−22.0 ± 4.5	−21.9 ± 4.5	−22.0 ± 6.3	−22.0 ± 5.1	−22.5 ± 4.1	−23.6 ± 5.6	−24.1 ± 7.1	−23.4 ± 5.7
**10**	−24.2 ± 5.9	−24.5 ± 7.7	−25.0 ± 6.9	−24.6 ± 6.8	−23.9 ± 5.4	−24.2 ± 5.4	−24.3 ± 7.6	−24.1 ± 6.1
**11**	−27.0 ± 5.9	−25.2 ± 6.3	−27.8 ± 7.6	−26.7 ± 6.6	−26.2 ± 7.6	−28.0 ± 6.9	−29.2 ± 9.1	−27.8 ± 7.9
**12**	−24.8 ± 7.1	−21.3 ± 6.3	−24.0 ± 4.8	−23.4 ± 6.2	−24.2 ± 6.6	−29.7 ± 7.5	−26.8 ± 7.5	−26.9 ± 7.5
**13**	−29.3 ± 6.0	−24.3 ± 7.3	−26.1 ± 8.8	−26.6 ± 7.6	−27.1 ± 8.8	−28.1 ± 7.1	−28.9 ± 7.4	−28.0 ± 7.7
**14**	−30.9 ± 7.8	−24.9 ± 9.0	−28.6 ± 7.7	−28.1 ± 8.5	−33.4 ± 8.4	−27.9 ± 8.1	−27.7 ± 9.9	−29.7 ± 9.1
**15**	−33.4 ± 8.3	−30.3 ± 8.8	−33.2 ± 10.1	−32.3 ± 9.1	−35.8 ± 8.4	−34.0 ± 8.5	−33.7 ± 10.8	−34.5 ± 9.2
**16**	−31.5 ± 7.8	−27.6 ± 9.0	−31.2 ± 7.6	−30.1 ± 8.2	−28.7 ± 7.8	−33.0 ± 7.2	−29.7 ± 8.8	−30.4 ± 8.1
**Global peak**	**−27.1 ± 2.9**	**−24.8 ± 4.4**	**−27.6 ± 4.9**	**−26.5 ± 4.2**	**−26.9 ± 3.4**	**−28.2 ± 3.4**	**−28.7 ± 4.1**	**−27.9 ± 3.7**
**Global mean**	**−26.4 ± 3.5**	**−23.9 ± 4.9**	**−26.8 ± 5.4**	**−25.7 ± 4.8**	**−26.6 ± 3.8**	**−27.6 ± 3.8**	**−27.7 ± 4.3**	**−27.3 ± 4.0**

All strain values are given in %.

**Table 6:** **Circumferential myocardial systolic strain values**

	**Men**				**Women**			
	**Tertile**				**Tertile**			
**Segment**	**1**	**2**	**3**	**All**	**1**	**2**	**3**	**All**
**1**	−22.2 ± 5.3	−19.3 ± 6.0	−20.7 ± 6.4	−20.7 ± 6.0	−20.7 ± 7.1	−21.0 ± 4.8	−22.5 ± 8.1	−21.4 ± 6.7
**2**	−19.9 ± 5.0	−20.4 ± 5.3	−18.6 ± 5.0	−19.7 ± 5.1	−22.7 ± 6.0	−19.3 ± 6.6	−23.9 ± 6.7	−21.9 ± 6.6
**3**	−20.8 ± 5.1	−18.6 ± 5.2	−20.5 ± 6.3	−20.0 ± 5.6	−22.6 ± 5.9	−22.1 ± 7.6	−23.9 ± 7.3	−22.9 ± 6.9
**4**	−17.5 ± 7.0	−15.2 ± 4.3	−21.2 ± 6.8	−17.9 ± 6.6	−17.7 ± 5.8	−18.6 ± 6.4	−19.5 ± 6.7	−18.6 ± 6.3
**5**	−20.8 ± 7.3	−18.4 ± 6.4	−24.5 ± 6.9	−21.2 ± 7.2	−21.1 ± 6.1	−22.8 ± 6.3	−27.4 ± 9.8	−23.5 ± 7.8
**6**	−23.3 ± 6.1	−19.7 ± 7.6	−28.4 ± 6.3	−23.7 ± 7.6	−24.1 ± 7.5	−28.5 ± 7.8	−27.7 ± 10.3	−26.7 ± 8.7
**7**	−19.8 ± 4.5	−18.2 ± 5.9	−22.2 ± 6.1	−20.1 ± 5.7	−21.4 ± 6.5	−22.6 ± 6.0	−21.3 ± 6.2	−21.8 ± 6.2
**8**	−17.9 ± 5.0	−18.9 ± 6.2	−18.3 ± 7.1	−18.4 ± 6.1	−18.8 ± 4.5	−18.1 ± 5.8	−19.8 ± 6.6	−18.9 ± 5.7
**9**	−17.5 ± 3.8	−16.8 ± 3.3	−17.9 ± 5.6	−17.4 ± 4.3	−18.3 ± 3.4	−18.8 ± 4.7	−19.0 ± 4.6	−18.7 ± 4.2
**10**	−18.2 ± 4.5	−18.0 ± 5.2	−19.0 ± 5.3	−18.4 ± 5.0	−18.6 ± 4.2	−18.5 ± 5.2	−17.9 ± 6.0	−18.3 ± 5.1
**11**	−20.9 ± 5.2	−18.5 ± 4.7	−21.4 ± 5.1	−20.3 ± 5.1	−20.7 ± 6.7	−22.4 ± 7.2	−23.5 ± 7.8	−22.2 ± 7.2
**12**	−20.2 ± 5.7	−16.3 ± 5.5	−19.3 ± 4.4	−18.7 ± 5.4	−20.3 ± 5.3	−25.3 ± 7.4	−22.0 ± 6.5	−22.5 ± 6.7
**13**	−22.3 ± 5.5	−17.8 ± 5.3	−19.8 ± 6.7	−20.0 ± 6.1	−21.3 ± 7.7	−21.9 ± 5.8	−21.7 ± 6.4	−21.6 ± 6.6
**14**	−25.7 ± 6.2	−19.4 ± 6.9	−23.1 ± 7.3	−22.7 ± 7.2	−27.3 ± 6.1	−22.1 ± 7.9	−23.0 ± 7.6	−24.2 ± 7.5
**15**	−27.4 ± 7.4	−23.9 ± 6.9	−26.1 ± 8.4	−25.7 ± 7.6	−30.8 ± 8.1	−27.2 ± 7.1	−27.0 ± 8.6	−28.4 ± 8.1
**16**	−23.7 ± 7.0	−20.0 ± 6.9	−22.9 ± 6.5	−22.2 ± 6.9	−23.6 ± 6.9	−25.9 ± 5.9	−21.9 ± 6.6	−23.8 ± 6.6
**Global peak**	**−21.1 ± 2.1**	**−18.7 ± 3.2**	**−21.4 ± 3.8**	**−20.4 ± 3.3**	**−21.9 ± 3.2**	**−22.2 ± 3.0**	**−22.6 ± 3.4**	**−22.2 ± 3.2**
**Global mean**	**−20.4 ± 2.6**	**−17.9 ± 3.5**	**−20.6 ± 4.2**	**−19.6 ± 3.7**	**−21.5 ± 3.5**	**−21.4 ± 3.3**	**−21.5 ± 3.7**	**−21.4 ± 3.5**

All strain values are given in %.

**Table 7:** **Longitudinal endocardial systolic strain values**

	**Men**				**Women**			
	**Tertile**				**Tertile**			
**Segment**	**1**	**2**	**3**	**All**	**1**	**2**	**3**	**All**
**1**	−22.8 ± 9.1	−17.7 ± 8.6	−18.0 ± 9.4	−19.5 ± 9.2	−29.6 ± 9.6	−25.4 ± 9.4	−25.0 ± 10.0	−26.7 ± 9.7
**2**	−25.1 ± 10.0	−25.7 ± 11.0	−22.5 ± 9.6	−24.4 ± 10.2	−28.3 ± 8.9	−24.0 ± 8.7	−23.6 ± 8.5	−25.3 ± 8.9
**3**	−20.3 ± 6.6	−21.7 ± 9.0	−21.7 ± 8.9	−21.2 ± 8.1	−21.0 ± 7.3	−23.9 ± 10.4	−19.3 ± 6.1	−21.5 ± 8.3
**4**	−24.4 ± 8.9	−19.8 ± 8.3	−22.1 ± 10.7	−22.1 ± 9.4	−25.7 ± 11.0	−27.9 ± 11.9	−28.6 ± 12.0	−27.4 ± 11.5
**5**	−23.8 ± 9.7	−29.5 ± 12.3	−30.5 ± 14.5	−27.9 ± 12.5	−32.9 ± 10.9	−35.1 ± 12.9	−34.0 ± 11.0	−33.9 ± 11.5
**6**	−28.9 ± 10.7	−29.1 ± 11.3	−32.8 ± 8.0	−30.3 ± 10.1	−29.6 ± 13.2	−32.5 ± 10.6	−36.2 ± 10.6	−32.8 ± 11.7
**7**	−30.7 ± 10.9	−22.0 ± 7.9	−24.5 ± 8.9	−25.8 ± 9.9	−30.5 ± 9.3	−29.9 ± 10.4	−31.3 ± 11.1	−30.5 ± 10.1
**8**	−22.5 ± 11.3	−21.8 ± 8.8	−18.2 ± 8.3	−20.8 ± 9.6	−25.9 ± 10.2	−23.2 ± 8.0	−21.6 ± 8.7	−23.6 ± 9.1
**9**	−17.3 ± 9.3	−16.9 ± 7.0	−16.2 ± 8.4	−16.8 ± 8.1	−16.8 ± 9.8	−17.9 ± 10.3	−13.8 ± 6.5	−16.1 ± 9.0
**10**	−17.0 ± 6.4	−15.8 ± 7.1	−15.0 ± 8.5	−16.0 ± 7.3	−18.8 ± 8.1	−17.4 ± 9.1	−18.1 ± 7.9	−18.1 ± 8.3
**11**	−18.1 ± 9.1	−17.7 ± 8.2	−22.3 ± 8.0	−19.3 ± 8.6	−21.5 ± 9.5	−26.6 ± 11.1	−20.8 ± 10.7	−22.9 ± 10.6
**12**	−18.6 ± 10.6	−19.9 ± 10.0	−21.2 ± 11.3	−19.9 ± 10.6	−21.8 ± 11.0	−26.0 ± 11.9	−24.8 ± 10.0	−24.2 ± 11.0
**13**	−26.7 ± 7.9	−23.2 ± 9.5	−30.0 ± 9.2	−26.6 ± 9.2	−25.3 ± 8.6	−28.4 ± 7.6	−29.2 ± 10.4	−27.6 ± 9.0
**14**	−24.5 ± 7.5	−20.9 ± 5.9	−22.4 ± 5.8	−22.6 ± 6.5	−20.5 ± 6.9	−23.3 ± 5.9	−24.0 ± 8.1	−22.6 ± 7.1
**15**	−22.2 ± 9.0	−22.0 ± 8.8	−24.8 ± 8.5	−23.0 ± 8.8	−22.0 ± 9.0	−22.5 ± 11.0	−23.0 ± 9.5	−22.5 ± 9.8
**16**	−20.1 ± 7.1	−14.6 ± 4.8	−20.3 ± 7.0	−18.3 ± 6.9	−19.3 ± 6.7	−19.6 ± 7.1	−17.4 ± 6.7	−18.8 ± 6.8
**Peak 2ch**	−23.6 ± 4.5	−19.7 ± 4.1	−22.2 ± 4.1	−21.8 ± 4.5	−24.8 ± 4.4	−25.8 ± 5.1	−26.7 ± 4.5	−25.8 ± 4.7
**Peak 3ch**	−23.0 ± 4.5	−22.0 ± 4.9	−23.2 ± 4.6	−22.7 ± 4.6	−24.6 ± 3.9	−27.5 ± 5.6	−24.3 ± 4.8	−25.4 ± 4.9
**Peak 4ch**	−20.1 ± 4.1	−19.4 ± 5.3	−20.9 ± 4.7	−20.1 ± 4.7	−21.2 ± 5.5	−21.8 ± 5.5	−20.8 ± 5.8	−21.3 ± 5.5
**Mean 2ch**	−22.1 ± 4.4	−17.9 ± 4.7	−19.7 ± 4.1	−19.9 ± 4.7	−23.1 ± 4.7	−24.2 ± 5.5	−24.9 ± 5.1	−24.1 ± 5.1
**Mean 3ch**	−20.8 ± 4.5	−20.3 ± 4.7	−21.1 ± 5.5	−20.7 ± 4.8	−23.0 ± 4.0	−25.8 ± 5.3	−22.6 ± 5.1	−23.8 ± 4.9
**Mean 4ch**	−17.9 ± 4.2	−17.6 ± 5.6	−19.2 ± 5.6	−18.2 ± 5.2	−19.0 ± 5.3	−19.9 ± 5.9	−19.1 ± 5.7	−19.3 ± 5.6
**Global peak**	**−22.7 ± 3.4**	**−21.1 ± 3.5**	**−22.8 ± 3.2**	**−22.2 ± 3.4**	**−24.4 ± 3.4**	**−25.2 ± 2.8**	**−24.4 ± 2.4**	**−24.6 ± 2.9**
**Global mean**	**−20.2 ± 3.5**	**−18.6 ± 3.8**	**−20.0 ± 3.7**	**−19.6 ± 3.7**	**−21.7 ± 3.5**	**−23.3 ± 3.6**	**−22.2 ± 3.6**	**−22.4 ± 3.6**

All strain values are given in %.

**Table 8:** **Longitudinal myocardial systolic strain values**

	**Men**				**Women**			
	**Tertile**				**Tertile**			
**Segment**	**1**	**2**	**3**	**All**	**1**	**2**	**3**	**All**
**1**	−20.0 ± 8.2	−16.7 ± 8.4	−15.8 ± 9.3	−17.5 ± 8.7	−27.3 ± 9.1	−23.6 ± 8.9	−22.4 ± 9.6	−24.4 ± 9.3
**2**	−24.3 ± 7.8	−25.8 ± 10.6	−24.1 ± 8.5	−24.7 ± 9.0	−27.4 ± 8.2	−25.0 ± 7.8	−24.2 ± 7.7	−25.5 ± 7.9
**3**	−20.7 ± 6.6	−20.3 ± 8.0	−20.6 ± 7.2	−20.5 ± 7.2	−22.2 ± 7.6	−23.8 ± 10.4	−19.1 ± 6.9	−21.7 ± 8.5
**4**	−22.9 ± 8.0	−19.6 ± 8.1	−21.9 ± 9.8	−21.4 ± 8.6	−25.2 ± 10.7	−27.6 ± 11.0	−27.5 ± 10.6	−26.8 ± 10.7
**5**	−24.2 ± 9.8	−28.8 ± 11.6	−30.0 ± 13.4	−27.7 ± 11.8	−33.8 ± 10.6	−34.3 ± 13.0	−33.9 ± 9.3	−34.0 ± 10.9
**6**	−28.9 ± 9.8	−28.0 ± 10.6	−31.1 ± 8.0	−29.4 ± 9.4	−30.3 ± 13.0	−31.8 ± 10.2	−36.2 ± 11.0	−32.9 ± 11.6
**7**	−27.8 ± 9.4	−19.4 ± 6.1	−21.7 ± 8.2	−23.0 ± 8.7	−28.0 ± 8.8	−27.6 ± 9.8	−28.5 ± 10.4	−28.0 ± 9.6
**8**	−20.3 ± 8.7	−20.7 ± 7.8	−18.3 ± 6.5	−19.8 ± 7.7	−23.3 ± 8.3	−21.4 ± 7.0	−19.9 ± 7.2	−21.5 ± 7.6
**9**	−16.8 ± 8.5	−15.6 ± 6.8	−15.6 ± 7.7	−16.0 ± 7.6	−17.3 ± 9.9	−19.6 ± 8.7	−13.3 ± 6.2	−16.6 ± 8.7
**10**	−15.0 ± 5.9	−14.5 ± 6.1	−13.8 ± 7.2	−14.4 ± 6.3	−16.8 ± 7.6	−16.2 ± 8.9	−15.5 ± 6.0	−16.1 ± 7.4
**11**	−18.2 ± 8.9	−17.6 ± 7.7	−21.7 ± 7.3	−19.1 ± 8.1	−21.5 ± 9.0	−25.3 ± 10.4	−20.3 ± 10.4	−22.3 ± 10.0
**12**	−19.0 ± 10.6	−19.4 ± 8.9	−19.0 ± 11.5	−19.1 ± 10.3	−21.8 ± 9.1	−25.2 ± 11.8	−23.3 ± 8.4	−23.4 ± 9.8
**13**	−23.0 ± 6.9	−18.2 ± 7.9	−25.0 ± 7.3	−22.0 ± 7.8	−21.7 ± 7.4	−23.5 ± 7.0	−24.8 ± 9.9	−23.3 ± 8.1
**14**	−19.9 ± 6.3	−17.0 ± 5.0	−18.3 ± 4.7	−18.4 ± 5.5	−17.0 ± 5.4	−19.1 ± 5.5	−19.3 ± 6.1	−18.4 ± 5.7
**15**	−18.2 ± 6.5	−16.2 ± 6.9	−19.3 ± 6.4	−17.9 ± 6.6	−18.4 ± 7.5	−17.2 ± 9.7	−18.0 ± 8.2	−17.9 ± 8.4
**16**	−16.4 ± 5.6	−11.4 ± 3.8	−15.1 ± 4.7	−14.3 ± 5.2	−15.7 ± 5.4	−15.6 ± 6.0	−13.7 ± 5.9	−15.0 ± 5.8
**Peak 2ch**	−20.8 ± 3.7	−17.1 ± 3.5	−19.4 ± 3.8	−19.1 ± 4.0	−22.4 ± 4.3	−23.1 ± 5.3	−23.6 ± 4.1	−23.0 ± 4.6
**Peak 3ch**	−21.1 ± 3.7	−20.5 ± 4.8	−21.7 ± 4.3	−21.1 ± 4.3	−23.0 ± 3.4	−25.5 ± 5.4	−22.6 ± 4.4	−23.7 ± 4.6
**Peak 4ch**	−19.0 ± 4.0	−17.6 ± 4.5	−18.9 ± 4.4	−18.5 ± 4.3	−20.5 ± 5.1	−20.9 ± 5.2	−19.6 ± 5.7	−20.3 ± 5.3
**Mean 2ch**	−19.2 ± 3.6	−15.4 ± 4.1	−17.2 ± 3.7	−17.3 ± 4.1	−20.7 ± 4.3	−21.6 ± 5.7	−22.0 ± 4.5	−21.4 ± 4.8
**Mean 3ch**	−19.1 ± 3.5	−18.8 ± 4.6	−19.8 ± 5.0	−19.2 ± 4.4	−21.5 ± 3.3	−24.0 ± 5.1	−21.1 ± 4.6	−22.2 ± 4.5
**Mean 4ch**	−16.9 ± 3.9	−15.9 ± 4.9	−17.2 ± 4.9	−16.7 ± 4.6	−18.4 ± 4.9	−18.9 ± 5.7	−17.8 ± 5.6	−18.4 ± 5.3
**Global peak**	**−21.0 ± 3.0**	**−19.3 ± 3.2**	**−20.8 ± 3.0**	**−20.4 ± 3.1**	**−23.0 ± 3.1**	**−23.4 ± 2.9**	**−22.4 ± 2.0**	**−22.9 ± 2.7**
**Global mean**	**−18.4 ± 2.9**	**−16.7 ± 3.5**	**−18.1 ± 3.3**	**−17.7 ± 3.3**	**−20.2 ± 3.1**	**−21.5 ± 3.7**	**−20.3 ± 3.3**	**−20.7 ± 3.4**

All strain values are given in %.

### Correlation between global peak and mean systolic strain values

The correlation between the global peak and mean systolic strain values were 0.97 for the radial, 0.96 for the circumferential endocardial, 0.98 for the circumferential myocardial, 0.87 for the longitudinal endocardial and 0.89 for the longitudinal myocardial strain. The radial early diastolic strain rate showed a correlation of 0.90, the circumferential endocardial early diastolic strain rate of 0.96, the circumferential myocardial early diastolic strain rate of 0.97, the longitudinal endocardial early diastolic strain rate of 0.90 and the longitudinal myocardial early diastolic strain rate of 0.90 between global peak and mean values.

### Observer agreement and variability

FTI allowed for reproducible quantification of radial, circumferential and longitudinal systolic strains, showing intra- and interobserver coefficients of variation of 7.9% and 10.0% for radial strain, −4.8% and −5.7% for circumferential endocardial strain and −4.3% and -4.8% for longitudinal endocardial strain, respectively. On a segmental level, the variations were higher yielding 11.4% and 11.5% for radial strain, −13.3% and −15.3% for circumferential endocardial strain and −16.9% and −21.1% for longitudinal endocardial strain. The detailed analysis of the segmental peak endocardial systolic strain values showed the best coefficient of variation in long axis views in the midventricular anterior segment (−11.6%) and the worst in the apical-septal segment (-33.1%) of the 3ch; the best and worst values in short axis orientation were found in the midventricular anteroseptal segment (-12.8%) and the basal anteroseptal segment (−22.5%). The coefficients of variations of all segments are given in Table 9.

**Table 9:** **Segmental reproducibility of systolic strain measurements**

**View**	**Segment**	**Coefficient of variation**
**2ch**	1	−23.5
	4	−20.3
	7	−11.6
	10	−25.3
	13	−18.4
	15	−19.7
**3ch**	2	−17.4
	5	−23.1
	8	−23.8
	11	−19.7
	14	−33.1
	16	−18.4
**4ch**	3	−19.8
	6	−21.0
	9	−16.3
	12	−19.1
	14	−17.7
	16	−30.0
**basal SAX**	1	−16.6
	2	−22.5
	3	−13.9
	4	−19.0
	5	−12.8
	6	−15.8
**mid. SAX**	7	−15.7
	8	−12.8
	9	−15.1
	10	−17.8
	11	−13.9
	12	−15.7
**apical SAX**	13	−17.0
	14	−15.9
	15	−14.9
	16	−18.9

The coefficient of variation is given in %.

mid: midventricular.

**Figure 4:**
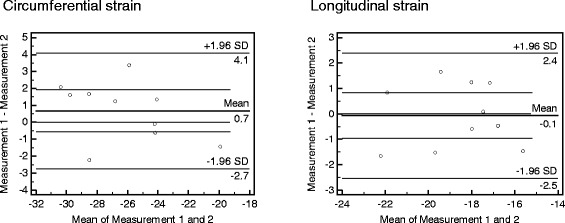
**Bland-Altman plots of the interstudy reproducibility.** Mean circumferential and longitudinal endocardial systolic strain values of ten subjects who underwent two consecutive CMR examinations. The time difference between both scans was 4.1 ± 3.5 days. The Bland-Altman plots include the line of equality and the lines of the 95% confidence interval of mean of differences.

The interstudy reproducibility test of ten subjects, who underwent two consecutive CMR examinations in 4.1 ± 3.5 days, yielded coefficients of variation of −4.8% for circumferential and −4.5% for longitudinal endocardial mean systolic strains. Bland-Altman plots are provided in Figure 4.

## Discussion

Our study demonstrates the ability of a CMR-based FTI algorithm to assess global and regional LV function in a large population of meticulously selected healthy subjects.

Objective and reproducible methods for the quantification of the myocardial function are of great clinical importance for patient management, therapy monitoring and outcome studies. The quantification using two-dimensional strain with echocardiography has nowadays emerged as an accurate predictor of clinical outcomes in various cardiac diseases [4,16,17]. However, echocardiography for strain assessment is generally associated with problems that are related to image quality, observer dependency, signal noise and angle dependency. Furthermore, a significant amount of segments may not be trackable due to impaired image quality [18,19]. Hence, tomographic imaging modalities like CMR may be a clinically valuable alternative to overcome such shortcomings. The current reference standard for myocardial deformation imaging in CMR is tagging. Previously, we have shown that another technique, SENC, is a highly reproducible method for wall motion quantification compared to MR tagging and has prognostic impact especially for patients with coronary artery disease [5]. A major drawback of both methods, however, is the need for extra CMR sequences that have to be acquired additionally, leading to a prolonged scanning time. Due to the post-processing approach of FTI, no additional scans are needed to be performed. Additionally, it allows the retrospective analysis of pre-acquired SSFP datasets. FTI itself provides a two-dimensional deformation analysis of the myocardium, which was originally designed for echocardiographic image analysis and has been previously validated in experimental and clinical settings [10,15]. Recently, FTI has been applied to regular cine SSFP sequences [10,20]. Of note, a good correlation of the FTI technique with CMR tagging has been already previously reported [10,11]. Thus, cardiac strain can be assessed without the need for additional CMR scans. It is furthermore independent of CMR vendors as well as of field strengths of 1.5 and 3 Tesla [7]. In the current study, at least 95% of the segments were assessable due to the robustness of the applied algorithm. Therefore, FTI offers the outstanding opportunity to perform all measurements using one imaging modality and to receive multi-dimensional information about the LV deformation pattern. “Tag fading”, which is a common technical problem in MR tagging due to the fading T1 signal of the tagging grids preventing the analysis of diastolic wall motion quantification, can be circumvented with FTI. Thus, FTI allows the quantification of the strains as well as the strain rates throughout the whole cardiac circle. As the FTI algorithms uses standard SSFP images and can be employed independently from MR vendor or field strength, CMR studies become comparable between different institutions which allows for multicenter studies. Consequently, the UK Biobank’s Cardiovascular MRI Advisory Group considers to replace tagging sequences by feature tracking [21].

Currently, further investigations, which compare the values derived from different types of CMR scanners directly, are needed.

### Age and gender dependency of cardiac deformation

In our study we employed FTI in a large population of healthy volunteers with a broad age range. Our inclusion criteria were based not only on their clinical history and examination, but also on extensive biochemical characterizations and CMR stress tests.

Male healthy subjects showed significantly lower circumferential and longitudinal strains resulting in less negative values whereas the radial strain featured higher values than women. In a study on 145 healthy volunteers Augustine et al. found similar gender differences [11]. Yet, the discrepancy regarding the circumferential strain in their study showed only a tendency whereas in our study it reached significance. The circumferential and longitudinal early diastolic strain rates were significantly lower in men than in women.

Interestingly, only the radial strain and the strain rates featured a significant age-dependency. With increasing age, the end-diastolic and end-systolic volumes decline clearly, whereas the LV mass remains constant or decreases slightly [22]. Furthermore, the ejection fraction and the mass-to-volume ratio increase. The age-related stiffness of the LV and the decline of the diastolic function could need to be compensated by an increased systolic wall thickening, which may explain the increasing radial strain with age.

Due to the age- and gender-related differences, we propose the use of specific reference values to avoid false positive or negative results especially in borderline cases.

Reference values from prior studies were derived from images acquired on Siemens or GE scanners [10,11] whereas our SSFP datasets were acquired on a Philips system. In a small study, Schuster et al. demonstrated that the results of FTI measurements derived from SSFP images obtained in a 1.5 T and a 3 T MR scanner were similar [7]. However, in future studies the vendor independency of the FTI algorithm for CMR has to be proven.

For clinical routine, fast and reproducible approaches are required. Therefore, we additionally investigated the role of the mean strain curves and their relation to age and gender. The correlation between the global peak and the mean systolic strain values was excellent. Especially the strains acquired in short axis views featured high correlation coefficients between 0.96 and 0.98.

### Study limitations

FTI algorithms are inherently dependent on image quality and endocardial border definition. For our analysis, we had to exclude some segments (short axis: 3.8%, long axis: 5.0%) because of suboptimal tracking. This was mainly due to poor endocardial or epicardial definition throughout the cardiac cycle, mostly because of prominent papillary muscles or epicardial structures. Additionally, since the FTI software does not supply a direct feedback on tracking quality, the global peak and mean strain values showed considerably lesser variances than the segmental values. As the segmental values had a considerably lower intra- and interobserver reproducibility, the use of the global peak and mean strain values is preferable in clinical routine for a robust delineation between physiological and pathological ventricular deformation. Yet, global values do not fully reflect the degree of regional wall motion abnormalities. For the assessment of regional deformation deficits, the use of the provided segmental strain values may be necessary although their diagnostic reliability is lower due their higher variability and lower reproducibility.

In the future, novel innovative parameters derived from advanced algorithms, as for example the global longitudinal strain (GLS), may offer additional and robust measurements with a high discriminatory value between normal and early dysfunctional myocardial wall motion patterns.

In this study, the FTI algorithm was applied to standard SSFP datasets featuring at least 35 phases per cardiac cycles. Thus, the temporal resolution is lower than in echocardiography, which might by relevant especially in the assessment of strain rates for the diastolic function.

Lastly, the parameters assessed by FTI were not compared to MR tagging or SENC, but a close correlation of these two methods has already been demonstrated before and was beyond the scope of this investigation.

## Conclusions

Our study provides reference values for cardiac deformation derived from a large population of healthy subjects using a novel feature tracking algorithm with high reproducibility. These values may be used for the evaluation of myocardial function or early onset of dysfunction in a clinical routine setting. Cardiac systolic strain values and diastolic strain rates differed partly significantly with respect to gender and to age, so that the employment of specific reference values is recommendable. In the future, further studies are needed to show the ability of FTI for the early detection of myocardial disorders and the assessment of prognosis.
